# Clinical observation of anterior decompression bone grafting and internal fixation in the treatment of lower cervical dislocation and analysis of influencing factors of early postoperative complications

**DOI:** 10.4314/ahs.v25i2.32

**Published:** 2025-06

**Authors:** Yongjun Meng, Zhichao Gao, Xiaoliang Qian, Jian Luo, Xiaodong Chu

**Affiliations:** Department of Orthopedic Surgery, The First Hospital of Linping District, Hangzhou, 311000, Hangzhou, China

**Keywords:** Inferior cervical vertebra, Dislocation, Anterior decompression, Bone graft fusion internal fixation, Early complications

## Abstract

**Objective:**

To investigate the effect of anterior decompression with bone graft and internal fixation for dislocation of lower cervical vertebra, and to analyze the influencing factors of early postoperative complications.

**Methods:**

The retrospective study included 90 patients with lower cervical fractures and dislocations who received anterior decompression and internal fixation in our hospital from November 2018 to October 2021. Postoperative indexes, therapeutic effects, postoperative recovery of cervical and spinal functions and complications were observed. Univariate and multivariate Logistic regression analysis was conducted with early postoperative complications as the dependent variable.

**Results:**

All the 90 patients underwent surgical treatment successfully, the operation time was (85.11 ± 11.57) min, the intraoperative blood loss was (148.05±36.75) mL. Compared with preoperative results, Cobb Angle and anterior atlas space were significantly smaller at 6 months and 1 year after surgery (P < 0.05). No broken plate, broken nail and loose plate were observed in the patient one year after surgery, and the bone graft fusion was good. JOA score and ASIA score were significantly improved at 6 months and 1 year after surgery (P < 0.05). ASIA scores of patients with incomplete nerve injury were improved in different degrees 6 months and 1 year after surgery (P < 0.05). Of the 90 patients, 10 had related complications after surgery, with an incidence of 11.11%. The results of multivariate Logistic regression analysis indicated that the time from injury to operation and the longer operation time were both influencing factors of early postoperative complications (P < 0.05).

**Conclusion:**

Anterior cervical approach decompression bone grafting and internal fixation in the treatment of lower cervical fracture and dislocation has remarkable effects, which is helpful to promote the recovery of spinal nerve function. However, postoperative complications still exist, among which the time from injury to operation and the longer the operation time are both influencing factors of early postoperative complications, which should be paid attention to in clinic.

## Introduction

Fracture and dislocation of the lower cervical spine is a relatively common traumatic disease in orthopedics at present, mainly occurring in the 3rd ∼ 7th segment of the cervical spine. The main clinical manifestations are the abnormal sequence and reduced stability of the cervical spine, accompanied by spinal cord injury and compression, resulting in different degrees of spinal cord dysfunction and limb paralysis in patients^1^. Especially high spinal cord injury, if not timely and effective treatment, will lead to high paraplegia, disability and fatality rate is high, seriously threaten the life and health of patients. In current clinical studies, anterior cervical surgery has become a common surgical method in spinal surgery, and has been widely used for cervical trauma, cervical tumor or tuberculosis, cervical disc degenerative diseases, etc^2^-^3^. For patients with fracture and dislocation of the lower cervical spine, surgical operations are mainly used, such as decompression, bone grafting, internal fixation and other measures, with the purpose of restoring the normal sequence and physiological curvature of the cervical spine, relieving spinal cord compression, and rebuilding the stability of the cervical spine^4^-^5^. The internal fixation method of lower cervical dislocation has been controversial in clinic. Advantages of anterior fixation fusion include: simple approach, less trauma, high fusion rate, and recovery of cervical interbody height and physiological radians. Reindl et al. reported 41 cases of lower cervical dislocation, 8 of which were successfully reduced by anterior overdistraction^6^. Alireza et al. performed anterior cervical bone graft fusion and internal fixation on 36 patients with lower cervical dislocation, and there was one patient with small joint fracture and three patients with laminar fracture who had failed surgery^7^. Due to the complex anatomy of the anterior cervical spine, which involves many important structures and is difficult to operate, intraoperative and postoperative complications such as cerebrospinal fluid leakage, spinal cord injury, neck hematoma, and improper internal fixation are likely to occur^8^-^9^. Currently, there have been reports on the clinical effect of anterior decompression bone grafting and internal fixation in the treatment of lower cervical dislocation, but there have been few reports on the influencing factors of early postoperative complications. In this study, 90 patients with lower cervical fracture and dislocation received in our hospital were selected to explore the clinical effect of anterior decompression and bone grafting and internal fixation in the treatment of lower cervical fracture and dislocation, and the influencing factors of early postoperative complications were analyzed.

## Materials and methods

### Information of Participants

The retrospective study included 90 patients with lower cervical fractures and dislocations who received anterior decompression and internal fixation in our hospital from November 2018 to October 2021. There were 59 males and 31 females; The age ranged from 19 to 69 years old, with a mean of (40.23±7.51) years old. Causes of injury: 48 cases were injured by car accident, 21 cases were injured by heavy objects, 13 cases were injured by falling, 8 cases were injured by falling. Fracture level: 11 cases C3 ∼ C4, 23 cases C4 ∼ C5, 45 cases C5 ∼ C6, 11 cases C6 ∼ C7; The average time from injury to operation was 14 ∼ 48h (32.29±5.06) h. Inclusion criteria: 1 CT/X-ray/MRI examination showed acute fresh injuries in the lower single level cervical spine (C3∼C7), with nerve root compression or spinal cord and nerve symptoms; 2 Combined with unilateral or bilateral dislocation of articular process; 3 Incomplete nerve Injury, i.e. ASIA (American Spinal Injury Association) classification B to E; 4 ≥ 18 years old; 5 Understand and give informed consent to this research. Exclusion criteria: 1 Automatic reduction in minor fracture traction; 2 Patients with cervical spondylotic myelopathy, congenital spinal deformity and other cervical diseases; 3 Patients with a history of severe anterior and posterior cervical spine injuries; 4 Patients with ossification of the ligamentum flavum and the posterior longitudinal ligament; 5 Complicated with serious organic diseases (such as heart, liver, kidney, lung, etc.) or endocrine and hematopoietic diseases; 6 Patients with metabolic bone disease, severe infection or tuberculosis; 7 Poor compliance or mental disorders can not cooperate with the study; 8 Clinical data are not complete. The incidence of postoperative complications was reported to be about 15%, according to data from reference studies. Assuming that the incidence of postoperative complications in this study is 15%, it is expected that two variables of the multivariate regression model will be included in this study eventually, and the sample size will be calculated according to the mean number of variable events (EPV), EPV=10, sample size = number of included variables *EPV/incidence =2*10/15%=133 cases.

### Methods

After admission, all patients received conventional cervical spine CT/X-ray/MRI imaging examinations to confirm the diagnosis, and then underwent skull traction or other manipulations combined with intraoperative field manipulation reduction. At the same time, routine dehydrating agents, hormones, neurotrophic drugs and large amounts of methylprednisolone were given treatment, and surgery could be performed after the patient's vital signs were stable. All patients were treated with anterior decompression and internal fixation with bone graft. General or local anesthesia was used for endotracheal intubation. Patients were placed in the supine position (the shoulders could be properly raised according to individual differences) to maintain cranial traction. Conventional disinfection towel, with the help of C-arm fluoroscopic image to identify the damaged segment. With the injured vertebra as the center, an oblique incision was made on the right side, and the platysma muscle, superficial and middle layer of deep cervical fascia were cut open. The esophagus, trachea and thyroid were pulled medial, and the carotid vessels were pulled lateral. The deep cervical fascia was bluntly separated and the longus cervical muscle was separated. Then the anterior longitudinal ligament and annulus fibrosus were incised by C-arm fluoroscopy to determine the decompression area. The injured tissue was scraped with a curette, and then the anterior part of the fractured vertebra was removed with a bone biter, and the nucleus pulposus tissue, intervertebral disc tissue and hematoma in the spinal canal were removed. If the vertebral body needs to be removed, subtotal resection of the fractured vertebra or discectomy can be performed for complete decompression. The tension of the retractor was adjusted to restore the height and physiological curvature of the injured vertebral body, and then the upper edge of the lower vertebral body was pry to restore the dislocated vertebral body. After the cervical spine sequence returned to normal, it was taken from the cortical iliac block on three sides of the body, or titanium mesh with appropriate length was selected to be implanted into the bone groove under cervical traction. The retractor was removed, and an anterior cervical plate of appropriate length was placed in front of the vertebral body, which was fixed and locked by screws. Two screws were used to fix the vertebral body and lock the plate. Again, the C-arm was used to check the reduction. After the ideal reduction, drainage strips were placed and the incisions were sutured layer by layer.

### Observation Indicators

(1) Surgery-related indicators: surgical success, operation time and intraoperative blood loss of patients were collected.
(2) Treatment effect: The changes of Cobb Angle of cervical vertebra and atlantodont space (odontoid process and anterior tubercle of atlas) of patients before surgery, 6 months after surgery, and 1 year after surgery were collected, as well as the fusion of bone graft 1 year after surgery.
(3) Cervical spine function and spinal nerve function: The Japanese Orthopaedic Association (JOA) score^10^ was used to evaluate the cervical spine of patients before surgery, 6 months after surgery, and 1 year after surgery, respectively. The evaluation contents included cervical subjective symptoms, clinical signs, limits of daily activities, and bladder function. The total score ranged from 0 to 29 points, and the score was proportional to the cervical spine function. Changes in spinal nerve function of patients before surgery, 6 months after surgery, and 1 year after surgery were evaluated and analyzed using the American Spinal Cord Injury Association (ASIA) scale^11^ : 1 Grade A (complete injury) : No motor or sensory function is preserved below the nerve plane of the spinal cord injury, including S4-S5 in the sacral segment (sellar region). 2 Grade B (incomplete injury) : Sensory function is preserved below the nerve plane of the spinal cord injury, including S4-S5 in the sacral segment, but no motor function is preserved. 3 Grade C (incomplete injury) : Motor function is preserved below the spinal cord injury nerve plane, but more than half of the key muscle strength below the spinal cord injury nerve plane is less than grade 3.4 Grade D (incomplete injury) : Motor function is preserved below the spinal cord injury nerve plane, and at least half of the key muscle strength below the spinal cord injury nerve plane is equal to or greater than grade 3.5 Grade E (normal) : normal sensory and motor function. The therapeutic effect and scale evaluation were completed by outpatient, return visit, home follow-up or telephone follow-up.
(4) Early complications: The incidence of incision infection, tissue injury, pneumothorax and other complications were retrospectively collected and counted, and the influencing factors of early postoperative complications were analyzed.

### Statistical Methods

All data in this study were input by Excel 2007 table software and imported into SPSS 25.0 statistical tool for processing. The measurement data were described by normality test (±s) and compared by t test. Counting data were expressed as percentage and chi-square test was used. Logistic regression analysis was used for multivariate comparison, with the occurrence of early postoperative complications as the dependent variable and each related factor as the independent variable. α=0.05 was set, and P < 0.05 meant the difference was statistically significant.

## Results

### Surgery-related indicators of patients

**Figure 1:**
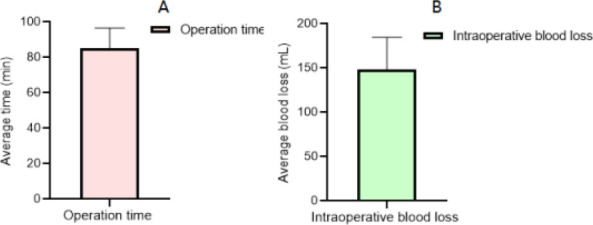
Surgical indicators of patients (A operation time, B intraoperative blood loss)

All patients successfully completed the operation, the operation time was (85.11 ±11.57) min, the intraoperative blood loss was (148.05±36.75) mL, as shown in Figure 1.

### Comparison of preoperative and postoperative changes of anterior atlas space and Cobb Angle in patients

The Cobb Angle and anterior atlas space were both smaller at 6 months and 1 year after surgery (P < 0.05). As shown in Table 1. No loose plates, broken plates or broken nails were observed in the patient one year after surgery, and the bone graft was well fused.

**Table 1:** Comparison of the changes of anterior atlantodentine space and Cobb Angle before and after surgery

time	Number of examples	Anterior atlantodontal space (mm)	Cobb Angle(°)
preoperative	90	6.35±0.82	31.37±4.69
6 months after surgery	90	2.46±0.50^*^	4.71±0.98^*^
1 year after surgery	90	2.03±0.26^*^	4.03±0.37^*^
*F*		1545.000	2842.000
*P*		<0.001	<0.001

**Note:** * indicates that compared with the operation, P < 0.05.

### Changes of JOA score and ASIA grading before and after surgery

JOA score and ASIA score were significantly improved at 6 months and 1 year after surgery (P < 0.05); ASIA scores of patients with incomplete nerve injury were improved in different degrees 6 months and 1 year after surgery (P < 0.05). Table 2.

**Table 2:** Changes of JOA score and ASIA grading before and after surgery (±s, points)

time	Number of examples	JOA Score	ASIA Score				

			A	B	C	D	E
preoperative	90	13.77±2.81	0 (0.00)	13 (14.44)	27 (30.00)	40 (44.44)	10 (11.11)
6 months after surgery	90	25.52±2.42	0(0.00)	6(6.67)	15(16.67)	42(46.67)	27(30.00)
1 year after surgery	90	26.50±2.03	0(0.00)	1(1.11)	3(3.33)	48(53.33)	38(42.22)
*F*		758.00			43.691		
*P*		<0.001			<0.001		

### Occurrence of early postoperative complications

**Figure 2:**
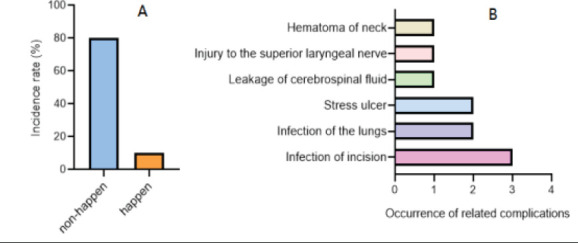
Occurrence of early postoperative complications (incidence of complications A, incidence of complications B)

Ten of the 90 patients had postoperative complications, with an incidence of 11.11%. There were 3 cases of incision infection, 2 cases of pulmonary infection, 2 cases of stress ulcer, 1 case of cerebrospinal fluid leakage, 1 case of superior laryngeal nerve injury, and 1 case of neck hematoma. These patients were cured after receiving symptomatic treatment such as adjusted use of antibiotics, anticoagulant and thrombolytic drugs, and supportive treatment during hospitalization. Are shown in Figure 2.

### Comparison of clinical data between the two groups

Ten cases with postoperative complications were taken as the group with complications, and 80 cases without complications were taken as the group without complications. The relevant data of the two groups were statistically processed, and the results showed that the time from injury to operation and operation time of the complication group were compared with those of the non-complication group (P < 0.05), and the other indicators of the two groups were compared (P > 0.05). Are shown in Table 3.

**Table 3:** Comparison of clinical data between the two groups

project	Complication group (n=10)	No complications group (n=80)	t/χ^2^	*P*
Gender (Male/female)	6/4	53/27	0.154	0.695
Age (years)	42.80±5.12	39.91±7.72	1.149	0.254
Time from injury to operation (h)	35.20±4.16	31.85±4.95	2.039	0.44
Method of anesthesia			0.415	0.520
General anesthesia	6(60.00)	56(70.00)		
Bureau of mass.	4(40.00)	24(30.00)		
Operative junction			0.738	0.864
C3 ∼ C4	1(10.00)	10(12.50)		
C4 ∼ C5	2(20.00)	21(26.25)		
C5 ∼ C6	5(50.00)	40(50.00)		
C6 ∼ C7	2(20.00)	9(11.25)		
Time of operation (min)	92.30±11.81	84.21±11.28	2.410	0.018
Intraoperative blood loss (mL)	155.40±44.55	147.13±35.89	0.669	0.505

### Multivariate Logistic regression analysis of early postoperative complications

Items with P < 0.05 in the above table 3 analysis were taken as independent variables, and whether patients had early postoperative complications was taken as dependent variables (1= yes, 0= no). Specific values were shown in Table 4. The results of multivariate Logistic regression analysis indicated that the time from injury to operation and the longer operation time were both influencing factors for early postoperative complications of patients (P < 0.05). As shown in Table 5.

**Table 4:** Assignment

Independent variable	Case of assignment
Injury to the time of surgery	Original value entry
Time of operation	Original value entry

**Table 5:** Multivariate Logistic regression analysis of early postoperative complications

variable	*β*	SE	Wals	*P*值	*OR* value	95%CI
Injury to the time of surgery	0.183	0.080	5.205	0.023	1.201	1.026∼1.405
Time of operation	0.094	0.040	5.475	0.019	1.099	1.015∼1.189
constant	-16.576	5.228	10.053	0.002	0.000	-

## Discussion

Fracture and dislocation of the lower cervical vertebra are easy to cause spinal cord injury and quadriplegia. If it occurs above the fifth cervical vertebra, respiratory and urinary infections, cardiovascular diseases, etc., may even lead to death^12^. The success rate of skull traction reduction or drug conservative treatment for lower cervical spine fractures and dislocations is low, and early surgical treatment is needed to achieve complete decompression, restore cervical spine sequence and curvature, and rebuild stability. At present, anterior cervical approach and posterior cervical approach are two common surgical approaches for the treatment of lower cervical fracture and dislocation, both of which can achieve good therapeutic effects^13^. Anterior decompression bone grafting and internal fixation can directly decompress the compressed vertebral body, reduce the impact on the spinal cord, ensure the stability of the spinal cord environment, and facilitate the recovery of spinal nerve function in patients^14^. In this study, by observing the influence of anterior decompression bone grafting and internal fixation on the dislocation of lower cervical vertebrae, it was found that the anterior cervical approach could shorten the operation time and reduce intraoperative bleeding. It was speculated that because the anterior cervical approach was simple and less traumatic, there was no need to cut off the muscles and important blood vessels during the operation. Studies have reported that postoperative Cobb Angle and horizontal displacement level of patients have no statistical significance. All the patients recovered well from fractures and dislocations after surgery, with low Cobb Angle and anterior atlantodental space, stable fixation and good bone graft fusion within 1 year after surgery, suggesting that the anterior cervical approach can promote fracture reduction and bone graft fusion, and has a good recovery effect on cervical spine sequence and physiological curvature, which may be related to plate support and tension fixation^15^-^16^.

Feng et al. reported a group of 21 patients with subcervical dislocation combined with TDH, who underwent anterior decompression distraction autogenous bone implantation and posterior reduction with long segment fixation fusion^17^. After 1-year follow-up, the patient's cervical kyphosis was completely recovered and neurological function was significantly improved. However, the major disadvantage of this technique is that long posterior fixation will sacrifice more cervical motion segments. Du et al. reported 17 cases of cervical dislocation patients with ASIA grade D or E with simple reduction and fixation by anterior traction and pry under the monitoring of evoked potential. Anatomical reduction was achieved in all patients, and neurological function was not deteriorated after surgery. Bone graft fusion was obtained in all patients 3 months after surgery, which was effective and worthy of clinical promotion^18^. The results of this study showed that the JOA scores of patients 6 months and 1 year after surgery were significantly improved compared with those before surgery, and ASIA scores of patients with incomplete nerve injury were improved to varying degrees (P < 0.05). It is suggested that anterior cervical approach decompression bone grafting and internal fixation in the treatment of lower cervical fracture and dislocation has a significant effect, which is helpful to promote the recovery of spinal nerve function. However, the results of this study showed that there were still 10 cases of early postoperative complications, with a total incidence of 11.11%, including 3 cases of incision infection, 2 cases of pulmonary infection, 2 cases of stress ulcer, 1 case of cerebrospinal fluid leakage, 1 case of superior laryngeal nerve injury, and 1 case of neck hematoma. During hospitalization, antibiotics, anticoagulant and thrombolytic drugs, supportive treatment and other symptomatic treatment were given and cured.

Many scholars have conducted retrospective studies on the causes and management of early complications after anterior surgery, and most of them believe that the complex local anatomical structure and perioperative management are related to the early complications to a certain extent, but the influencing factors have not been analyzed. In this study, multivariate analysis of the occurrence of early complications was further conducted, and it was found that the time from injury to operation and the longer the operation time were both influencing factors for the early postoperative complications of patients (P < 0.05). Most patients with lower cervical fracture and dislocation are in a state of stress, and the time from injury to operation is prolonged, which cannot be timely and effective treatment. With the further development of the disease, limb movement disorders and sensory disorders may occur, and even lower limb paralysis or high amputation may occur, which is not conducive to the prognosis of patients^19^. Traction and reduction should be performed on the patient in time after admission to reduce the compression and damage to the spinal cord and provide necessary time for the treatment of complications^20^. However, due to the complex and traumatic operation of anterior decompression and internal fixation with bone grafting, it is necessary to completely remove the pressurized intervertebral disc tissue, posterior ligament and bone mass, confirm complete decompression, and then perform subsequent operations^21^. If the intraoperative exposure time is too long, the affected area of the patient is likely to be infected, and the patients with the disease themselves have lower tolerance and resistance to the operation, thus increasing the risk of complications^22^-^23^. Therefore, clinically, for such patients, preoperative preparation should be made and perioperative treatment should be done as well as possible, so as to reduce the risk of postoperative complications.

## Conclusion

Anterior cervical approach decompression bone grafting and internal fixation in the treatment of lower cervical fracture and dislocation has remarkable effects, which is helpful to promote the recovery of spinal nerve function. However, postoperative complications still exist, among which the time from injury to operation and the longer the operation time are both influencing factors of early postoperative complications, which should be paid attention to in clinic. In this study, the number of samples is limited and the follow-up time is short, so the long-term effect is not discussed, and further analysis is needed to improve the scheme.
